# Visual Search of Mooney Faces

**DOI:** 10.3389/fpsyg.2016.00155

**Published:** 2016-02-12

**Authors:** Jessica E. Goold, Ming Meng

**Affiliations:** Department of Psychological and Brain Sciences, Dartmouth College, HanoverNH, USA

**Keywords:** face detection, attention, parallel search, Mooney image, object recognition

## Abstract

Faces spontaneously capture attention. However, which special attributes of a face underlie this effect is unclear. To address this question, we investigate how gist information, specific visual properties and differing amounts of experience with faces affect the time required to detect a face. Three visual search experiments were conducted investigating the rapidness of human observers to detect Mooney face images. Mooney images are two-toned, ambiguous images. They were used in order to have stimuli that maintain gist information but limit low-level image properties. Results from the experiments show: (1) Although upright Mooney faces were searched inefficiently, they were detected more rapidly than inverted Mooney face targets, demonstrating the important role of gist information in guiding attention toward a face. (2) Several specific Mooney face identities were searched efficiently while others were not, suggesting the involvement of specific visual properties in face detection. (3) By providing participants with unambiguous gray-scale versions of the Mooney face targets prior to the visual search task, the targets were detected significantly more efficiently, suggesting that prior experience with Mooney faces improves the ability to extract gist information for rapid face detection. However, a week of training with Mooney face categorization did not lead to even more efficient visual search of Mooney face targets. In summary, these results reveal that specific local image properties cannot account for how faces capture attention. On the other hand, gist information alone cannot account for how faces capture attention either. Prior experience facilitates the effect of gist on visual search of faces; making faces a special object category for guiding attention.

## Introduction

Faces capture our attention. Humans are able to saccade toward a face in as little as 100 ms, whereas it is difficult to saccade away from faces (Crouzet et al., 2010). Faces are also efficiently detected in visual search tasks (Hershler and Hochstein, 2005; Williams et al., 2005; cf. VanRullen, 2006; Doi and Ueda, 2007). It has been postulated that emergent properties in a scene are perceived before more intricate details are processed (Doi and Ueda, 2007). Because faces are detected so quickly, they may contain an emergent property that guides our attention in order to process informative social cues. Previous behavioral testing has demonstrated that humans can correctly detect a face in a scene displayed for as briefly as 12 ms, too quick for attention to be allocated to a specific location (Graham and Meng, 2011). Also, electrophysiological studies reveal that selective brain responses to faces occur at 100ms or less (Crouzet et al., 2010; Cauchoix et al., 2014). Prior research investigating which properties of a face capture our attention has focused on facial expressions (Williams et al., 2005) and direction of eye gaze (Doi and Ueda, 2007) amongst distractors with neutral facial expression or opposing eye gaze. However, category information has also been shown to guide attention (Yang and Zelinsky, 2009). Which properties of the category ‘face’ guide our attention remains highly controversial (Li et al., 2002; Rousselet et al., 2003; Evans and Treisman, 2005; Hershler and Hochstein, 2005; VanRullen, 2006; Palermo and Rhodes, 2007; Rossion and Caharel, 2011). Here we specifically evaluate the effects of gist information, individual features and amount of prior experience with the target faces on efficiency of detection.

**FIGURE 1:**
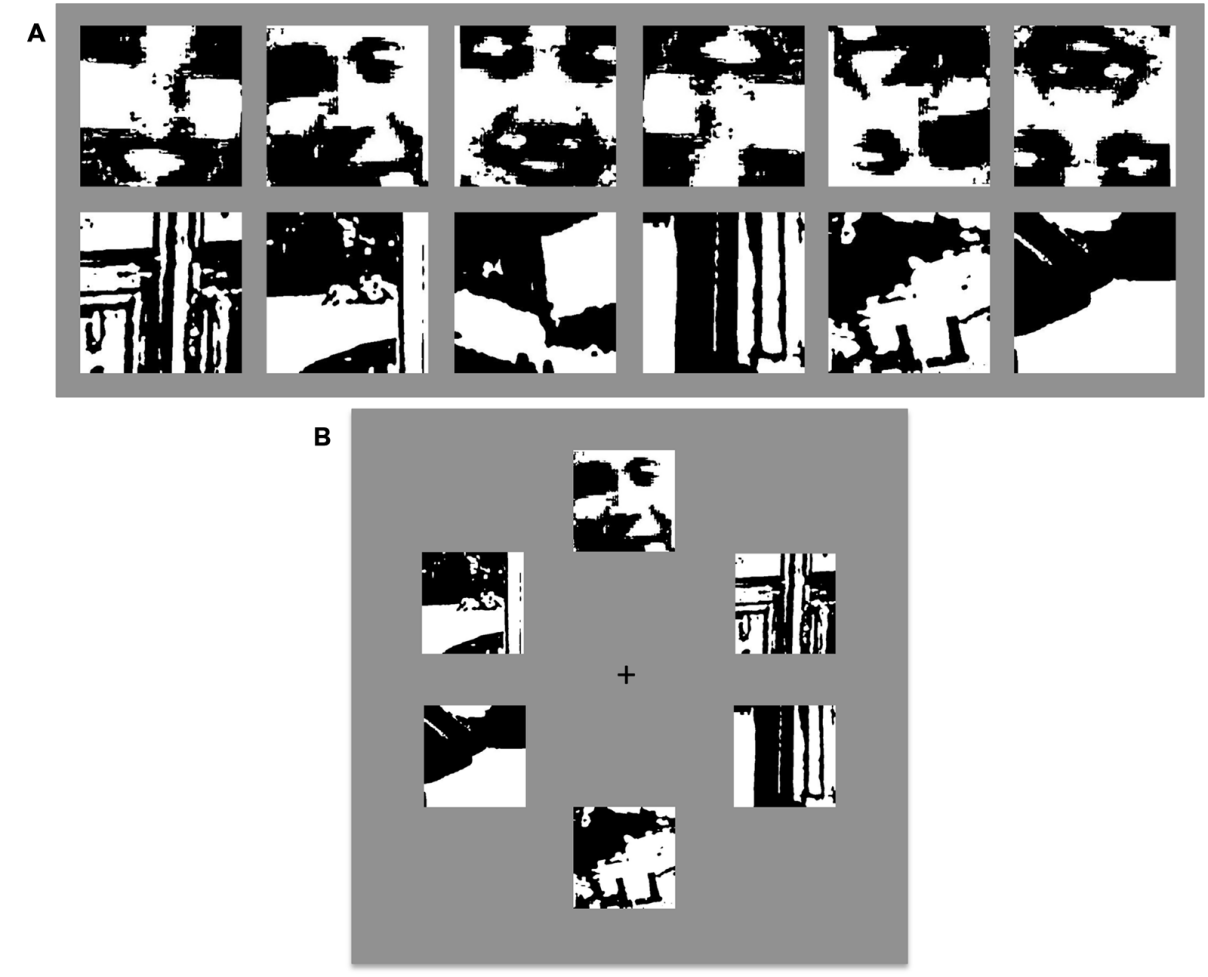
**Examples of Mooney Stimuli and Paradigm. (A)** Top row: Examples of Mooney face targets both upright (first three) and inverted (last three). Bottom row: Examples of distractor stimuli. **(B)** An example of a visual display containing a Mooney face target (at the top position) and 5 Mooney, non-face distractors.

The reverse-hierarchical theory of visual processing proposes that overall gist information is processed pre-attentively (Hochstein and Ahissar, 2002). Gist is considered to be the meaningful information one can extract in an instant (Oliva, 2005; Loschky and Larson, 2008) and this information guides attention to emergent properties of an image for further scrutiny (Hochstein and Ahissar, 2002). Mooney images (Mooney, 1957) are two-toned, ambiguous images made my manipulated a gray-scale image (**Figure 1A**). Although visually degenerated, upright Mooney faces share the same gist and configural information with normal face pictures. Mooney images are also controlled for low-level features and experience, making them an ideal candidate to investigate the role of gist information on face detection. Gist information in upright face images has been shown to be important in face detection. For instance, when a face target is inverted or scrambled, disrupting the gist of a face, search efficiency is destroyed and neural responses in face responsive areas are diminished (Brown et al., 1997; Hershler and Hochstein, 2005). Also, using continuous flash suppression (i.e., a flashing Mondrian pattern is presented to one eye, and a static image is presented to the other eye, causing a suppression effect of the static image), upright faces break through suppression faster than inverted faces (Jiang et al., 2007). Developmental research has further presented evidence that newborns attend to upright face patterns more than their inverted counterpart, suggesting an innate preference for the gist of a face (Morton and Johnson, 1991; Nelson, 2001). It has been hypothesized that face detection may occur through an innate and automatically faster subcortical route (Johnson, 2005). If this is the case, the gist of faces, which includes both social and emotion information, may be rapidly processed through the subcortical pathway. Thus, rapidness of face detection should then be independent of details of specific features.

However, the effects of image-level visual properties, such as spatial frequency and skin color, have also been implicated in affecting the efficiency of face detection. For example, VanRullen (2006) manipulated the amplitude spectrum of face images by replacing them with the amplitude spectrum of car images and destroyed search efficiency for faces, suggesting that the amplitude spectrum of the face underlies pre-attentive processing. It has also been reported that EEG activity correlating with image-level properties, such as face size, could be used to accurately categorize visual stimuli as faces within 94 ms of stimulus onset (Cauchoix et al., 2014). This suggests that individual feature information may be involved in guiding attention to faces for fast processing. Investigating visual search of Mooney faces would allow us to tease apart possible effects of gist information and individual features. If it is the gist information in a face that captures our attention, we should find efficient detection in Mooney face images regardless of manipulations to any residual low-level features.

Using Mooney images also allows us to examine how prior experience may modulate effects of gist information and individual features in rapid face detection. Recognition of Mooney images is known to be heavily modulated by top–down effects of prior experience (Dolan et al., 1997; Hsieh et al., 2010; Gorlin et al., 2012). The influences of being social animals and the tremendous amount of experience humans have with faces have been proposed to underlie the attention grabbing nature of faces (Diamond and Carey, 1986; Gauthier et al., 2000). Based on this hypothesis, it is expected that all categories of which an individual is an expert should have similar processing advantages to faces. Indeed, behavioral and neural effects similar to those found for faces have been found for objects of expertise. Diamond and Carey (1986) found that dog show judges had an inversion effect for dog breed recognition. Moreover, the fusiform face area (Kanwisher et al., 1997), an area of the lateral fusiform gyrus which responds to face stimuli more than other tested non-face stimuli, has been reported to positively respond to categories of expertise (Gauthier et al., 1999). However, it is not clear how visual experience may shape face processing (Le Grand et al., 2001a,b; Fine et al., 2003; Ostrovsky et al., 2006; Lorenzino and Caudek, 2015). Whereas perceptual learning of feature conjunctions is possible (Wang et al., 1994; Carrasco et al., 1998), large amounts of visual experience and eventually expertise with faces may also underlie efficient face detection and rapid face processing by enhancing the extraction of gist information from Mooney face images.

In summary, what properties of a face capture attention remains unclear. To address this question, here we conducted a series of three visual search experiments. Visual search is a classic psychophysical paradigm for investigating visual attention. A search is considered efficient when a target is detected independently of the number of distractors in the display. If a target is searched efficiently, it captures our attention (Treisman and Gelade, 1980). It has been postulated that efficient search is invoked when there is a single-feature difference between target and distractors. However, face images are searched very efficiently, despite the absence of a clear, distinctive single-feature difference between faces and non-face objects (Hershler and Hochstein, 2005; Yang et al., 2011). We further combined visual search with Mooney images. Using Mooney images allows for control of low-level features and experience while maintaining gist information, making it an advantageous tool for investigating the effect of gist on guiding attention. Moreover, based merely on local features, recognizing the object content in Mooney images is impossible. Therefore, holistic processing is necessary for recognizing Mooney faces (McKone, 2004; Farzin et al., 2009). If Mooney faces were searched efficiently, it would suggest that holistic, gist information of a face is enough to guide attention. On the other hand, if observers rely on image-level visual features to rapidly detect faces, searching for a Mooney face among non-face Mooney images would not be efficient. And lastly, if observers rely on conceptual knowledge and experience to rapidly detect faces, all searches would be inefficient unless prior information about the target was provided.

## Experiment 1

### Methods

#### Participants

Twenty-eight (18 female) students from Dartmouth College volunteered to participate in Experiment 1. All participants gave written, informed consent and had normal or corrected to normal visual acuity. All participants received course credit or were compensated for their time. Sample sizes were chosen in order to be comparable with that of other similar visual search studies (Wolfe, 1998; Tong and Nakayama, 1999). These procedures were approved by the Committee for the Protection of Human Subjects at Dartmouth College and conducted in accordance with the 1964 Declaration of Helsinki.

#### Materials and Procedure

A set of 50 gray-scale face images and 100 gray-scale non-face images were transformed into Mooney images for the experiment. The face images consisted of frontward facing, male and female faces, cropped to exclude hair and ears. The non-face images were cropped parts of scenes and objects. To create Mooney images, MATLAB with SHINE toolbox was used (Willenbockel et al., 2010). First, the median luminance of each gray-scale image was found. Next, the images were manipulated such that all of the pixels in the image with the median luminance value or higher were changed to white, and all of the pixels in the image with luminance values lower than the median were changed to black.

The experiment was coded using MATLAB with Psychtoolbox on a 21-inch Dell P1130 CRT monitor with a refresh rate of 85 Hz and spatial resolution of 1280 × 1024 pixels (Brainard, 1997). The visual search display was similar to a previously published design (Tong and Nakayama, 1999), with a black fixation cross in the center of a gray screen and 2, 4, or 6 images, positioned angularly around the fixation point at 30, 90, 150, 210, 270, and 330° (see **Figure 1B**). For trials with less than 6 stimuli, positions were randomly selected among the six options. Each image was ∼5° from the fixation point and subtended ∼5° of visual angle.

In a randomized mixed design, each participant was tested with 2400 trials composed of 1200 target-present trials (600 upright face targets and 600 inverted face targets) and 1200 target-absent trials (600 upright distractor image displays and 600 inverted distractor image displays). Each condition also had an equal number of trials with 2, 4, or 6 images in the display array. In the target-present trials, the search target was randomly chosen from the 50 Mooney face images (see examples in **Figure 1A** top row) and distractors were randomly chosen from the 100 Mooney non-face images (see examples in **Figure 1A** bottom row). In the target-absent trials, all images were randomly chosen from the 100 Mooney non-face images. No image was presented more than once in the same trial. Target and distractors were presented upright in the upright condition, and upside-down in the inverted condition. Participants were instructed to maintain fixation at the center of the screen and search for a face in each trial; pressing the ‘F’ key if a face was present, and the ‘J’ key if there was no face as quickly as possible while maintaining high accuracy. A tone played if an incorrect response was made. Trials ended following the participant’s response and instructions appeared asking to press the spacebar for the next trial in order to minimize possible position aftereffects from the previous trial. This also gave participants a chance to take a break after any trial if needed. Every 600 trials the experiment stopped and participants had to take a break before they could begin another 600 trials.

### Data Analysis

Accuracy rates for each condition were computed to examine the possibility of speed-accuracy trade-offs. Data analysis focused on the RTs of correctly answered trials. The trials containing the slowest 2.5% of RTs as well as the quickest 2.5% of RTs were trimmed off to exclude outliers. A three-way ANOVA was conducted with set size, inversion, and target presence as within-subject factors.

### Results

**FIGURE 2:**
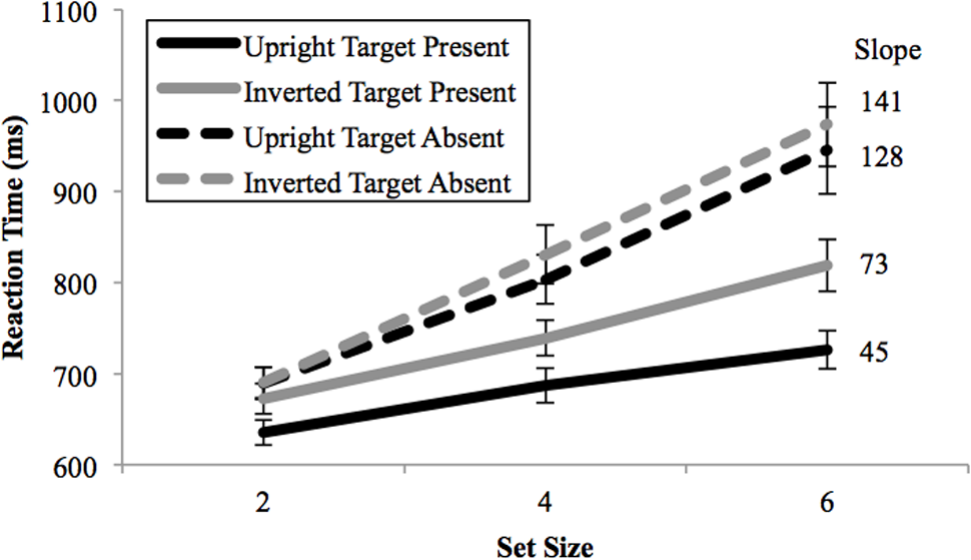
**Mean reaction times as a function of search array set size in Experiment 1.** Upright Mooney faces were searched more efficiently than inverted Mooney faces. Black lines = upright conditions; Gray lines = inverted conditions; Solid lines, target-present; dash lines, target-absent. Search reaction time slopes for each condition are shown in ms/item to the right of the corresponding lines. Error bars represent ± 1 *SEM*.

Accuracy rates ranged from 85.8 to 93.5% correct across all conditions. Overall, the averaged accuracy rate across subjects for upright trials (92.2%) was greater than inverted trials (88.5%), with no evidence of speed–accuracy trade-offs. For the correct trials, averaged RTs by set size for each condition are plotted in **Figure 2**. The three-way ANOVA revealed significantly faster RTs for upright than inverted trials (black lines vs. gray lines: *F*(1,27) = 75.17, *p* < 10^-10^, $\eta_{p}^{2}$ = 0.74), while target-present trials were significantly faster than target-absent trials (solid lines vs. dotted lines: *F*(1,27) = 43.91, *p* < 10^-6^, $\eta_{p}^{2}$ = 0.62). The effect of set size was also highly significant [*F*(2,54) = 73.84, *p* < 10^-11^, $\eta_{p}^{2}$ = 0.73], showing that the Mooney face targets were not searched efficiently. Significant interactions were found between inversion and target presence [*F*(1,27) = 49.29, *p* < 10^-7^, $\eta_{p}^{2}$ = 0.65], set size and target presence [*F*(2,54) = 37.31, *p* < 10^-11^, $\eta_{p}^{2}$ = 0.58] and inversion and set size [*F*(2,54) = 17.34, *p* < 10^-5^, $\eta_{p}^{2}$ = 0.39]. The three-way interaction between inversion, set size and target presence was not significant [*F*(2,54) = 1.06, *p* = 0.35, $\eta_{p}^{2}$ = 0.04].

These results demonstrate that gist information contributes significantly to rapid face detection but does not fully explain how faces capture attention. Upright Mooney face targets were detected more rapidly (635 vs. 672 ms at set size 2) and more efficiently than inverted search targets (45 ms/item vs. 73 ms/item). However, upright Mooney faces were detected with a significant main effect of set size (the black, solid line in **Figure 2** is not flat), suggesting the involvement of attention. Indeed, the search speed for Mooney face stimuli is less efficient than previous reports from a study using intact face pictures as search targets (Hershler and Hochstein, 2005). Given that image-level features were equalized to a great extent in Mooney images, it is possible that the presence of features specific to different intact face pictures may underlie faster detection resulting in efficient search in the previous study. If that were the case, some residual, non-equalized features in certain Mooney faces could then potentially enable them to be searched more efficiently than the others. To test this possibility, in Experiment 2 we used a block design with an individual Mooney face target for each block. If search efficiency were different for different Mooney face targets, it would suggest that specific individual-level features guide attention to enhance search efficiency. However, if all upright faces were searched with equal efficiency, it would suggest that those individual-level features are not used to rapidly differentiate face/non-face, since those features would not aid in search speed.

## Experiment 2

### Methods

#### Participants

Twenty-four (13 female) students from Dartmouth College volunteered to participate in Experiment 2. All participants had normal or corrected to normal visual acuity. All participants were unaware of the purpose of the experiment and had not participated in an experiment with the same set of images. All participants gave written, informed consent and received course credit or compensation for their time. These procedures were approved by the Committee for the Protection of Human Subjects at Dartmouth College and conducted in accordance with the 1964 Declaration of Helsinki.

#### Materials and Procedure

Six Mooney face target images were randomly selected from the 50 faces in Experiment 1 to be the targets in Experiment 2. Distractors were the same as in Experiment 1. One Mooney face target was used for each block. Each participant had the same six Mooney faces as the search targets, however, three of them were presented upright and the other three were inverted. Which three of the six were shown upright and which were shown inverted were counterbalanced between participants. To ensure that practice did not cause the search of upright Mooney faces to be faster than inverted Mooney faces, the first three blocks had upright Mooney faces as the targets, whereas the last three blocks had inverted Mooney faces as the targets. All of the distractors were upright regardless of whether the block had an upright or inverted target. As shown by Experiment 1, inversion of Mooney non-face images as the distractors did not have any effect. Each block contained 360 trials with 50% target-present trials and 50% target-absent trials.

The target was shown at the beginning of each block along with the same instructions from Experiment 1. Participants could study the target for as long as they liked before pressing any key to start the block. Trials were set up identically to Experiment 1. Participants were asked to take breaks between blocks, but they could also take a break before any trial if they wanted.

### Data Analysis

Accuracies were analyzed as in Experiment 1. Only the RTs of correctly answered trials were used, and the outliers were excluded using the same criteria as Experiment 1. A mixed-model four-way ANOVA was performed on the remaining RTs with set size, identity, inversion, and target presence as the factors. Next, two three-way ANOVAs were conducted on both the target-present and target-absent conditions separately, with set size, identity, and inversion as the three factors.

### Results

**FIGURE 3:**
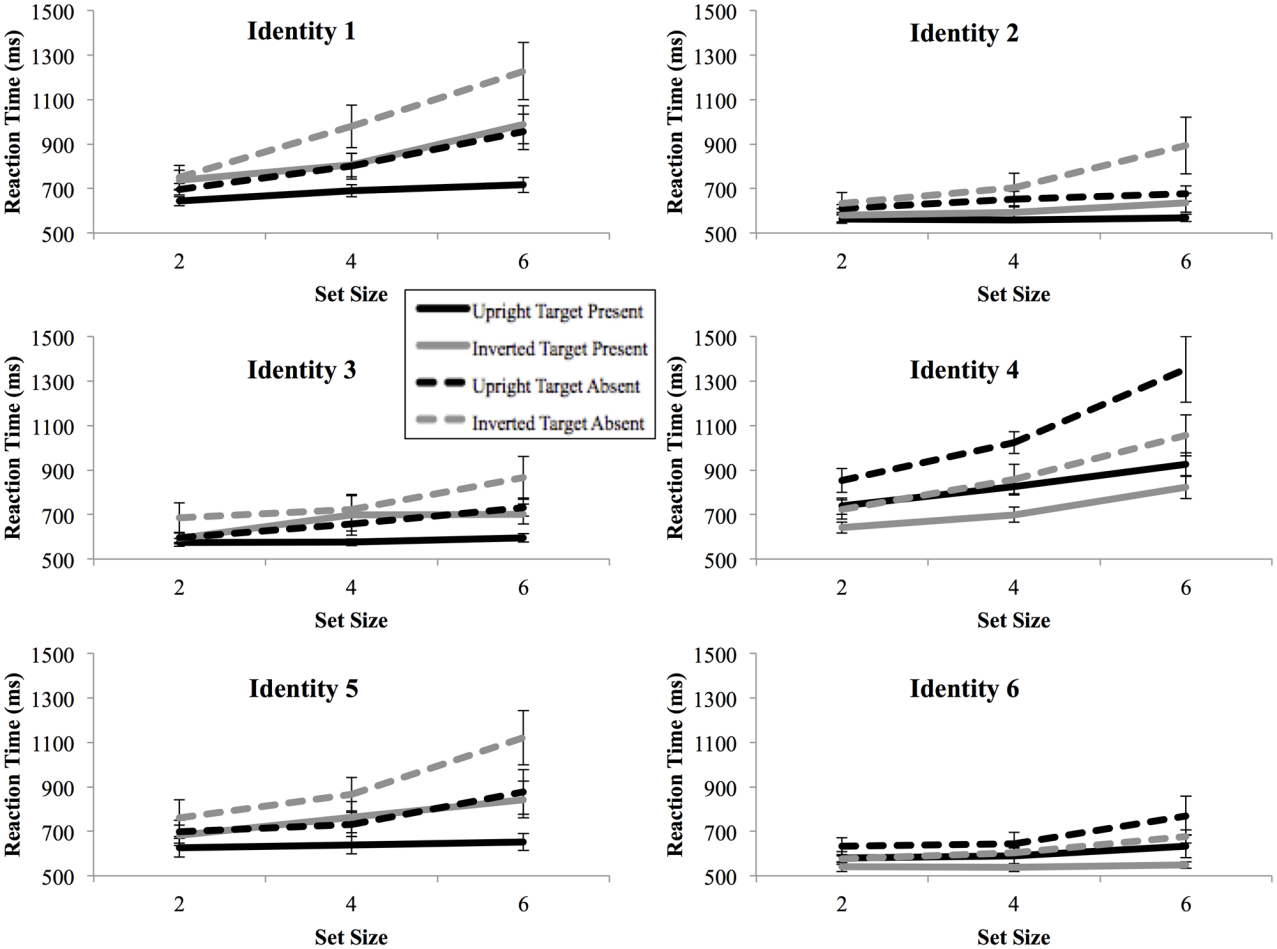
**Mean reaction times as a function of search array set size for each target image in Experiment 2.** Slightly different trends for the search reaction times are observed for different targets. Black lines, upright condition; gray lines, inverted conditions; Solid lines, target-present; dash lines, target-absent. Error bars represent ± 1 *SEM.*

The overall accuracy rate was high for both upright Mooney face conditions (97.1%) and inverted Mooney face conditions (96.5%), with no evidence of speed–accuracy trade-offs. **Figure 3** shows the RTs by set size for each identity and each condition. The four-way ANOVA on RTs revealed significant main effects of identity [*F*(5,50) = 27.78, *p* < 10^-12^, $\eta_{p}^{2}$ = 0.74], set size [*F*(2,20) = 51.67, *p* < 10^-8^, $\eta_{p}^{2}$ = 0.84], inversion [*F*(1,10) = 6.19, *p* < 0.05, $\eta_{p}^{2}$ = 0.38], and target presence [*F*(1,10) = 46.58, *p* < 10^-5^, $\eta_{p}^{2}$ = 0.82]. The interaction between identity and set size was also significant [*F*(10,100) = 20.82, *p* < 10^-13^, $\eta_{p}^{2}$ = 0.68], showing that some identities were searched more efficiently than others. Significant interactions were also found between set size and inversion [*F*(2,20) = 17.62, *p* < 10^-5^, $\eta_{p}^{2}$ = 0.64], identity and target presence [*F*(5,50) = 13.31, *p* < 10^-8^, $\eta_{p}^{2}$ = 0.57], and set size and target presence [*F*(2,20) = 31.84, *p* < 10^-7^, $\eta_{p}^{2}$ = 0.76]. All other interactions were not significant.

**FIGURE 4:**
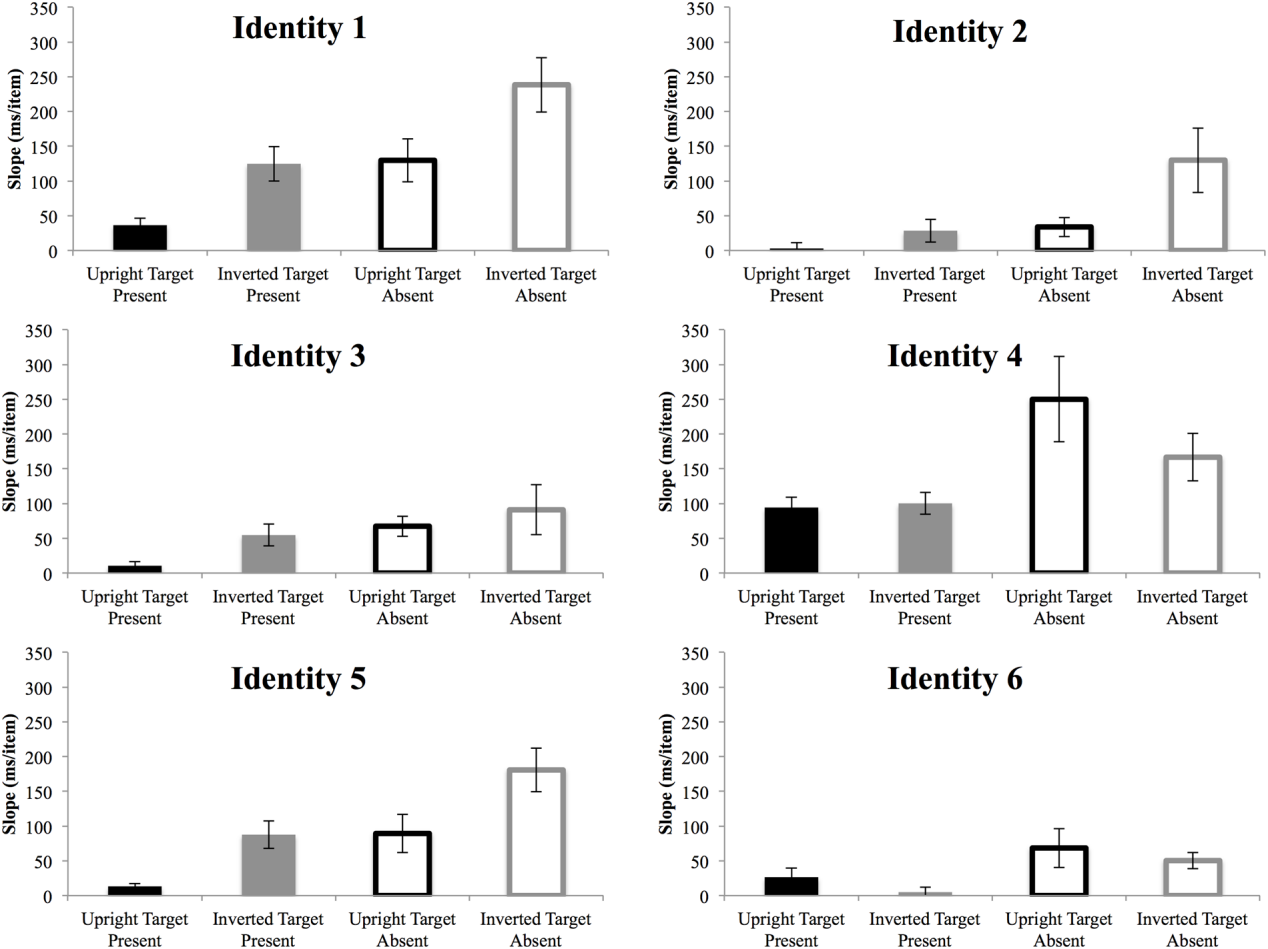
**Slopes of the reaction times as a function of search array set size for each target in Experiment 2.** While the efficiency of search varies for each target, the main effect of inversion is evident in the majority of targets. Black bar, upright; gray bar, inverted; filled bar, target-present; hollow bar, target-absent. Error bars represent ± 1 *SEM.*

For target-present trials, the three-way ANOVA revealed a significant main effect of identity [*F*(5,50) = 19.51, *p* < 10^-11^, $\eta_{p}^{2}$ = 0.66], set size [*F*(2,20) = 36.22, *p* < 10^-7^, $\eta_{p}^{2}$ = 0.78] and inversion [*F*(1,10) = 8.34, *p* < 0.05, $\eta_{p}^{2}$ = 0.46]. The interaction between identity and set size was significant [*F*(10,100) = 7.53, *p* < 10^-9^, $\eta_{p}^{2}$ = 0.43]. The interaction between set size and inversion [*F*(2,20) = 12.77, *p* < 10^-4^, $\eta_{p}^{2}$ = 0.56], and the three-way interaction between identity, set size and inversion were also significant [*F*(10,100) = 3.06, *p* < 0.05, $\eta_{p}^{2}$ = 0.24]. The three-way ANOVA on target-absent trials also revealed significant main effects of identity [*F*(5,50) = 31.87, *p* < 10^-14^, $\eta_{p}^{2}$ = 0.76] and set size [*F*(2,20) = 48.46, *p* < 10^-8^, $\eta_{p}^{2}$ = 0.83]. Significant interactions were found between identity and set size [*F*(10,100) = 12.67, *p* < 10^-10^, $\eta_{p}^{2}$ = 0.56] and between set size and inversion [*F*(2,20) = 6.49, *p* < 0.01, $\eta_{p}^{2}$ = 0.39]. Slopes for each identity and condition are further shown in **Figure 4**. Two of the upright face targets and one of the inverted face targets were searched efficiently, with slopes of less than 10 ms/item.

The search speed slopes for upright Mooney faces were significantly less than inverted Mooney faces. Since upright Mooney faces were the targets for the first three blocks and inverted Mooney faces were the targets for the last three blocks, these results are unlikely caused by any effect of practice. Moreover, if there had been an effect of practice, the RTs of trials at the end of blocks would have been different from the RTs at the beginning of blocks. No such effect was found. Taken together, as differentiating upright vs. inverted Mooney faces is impossible with merely local feature information, the results of Experiments 1 and 2 have demonstrated that gist information contributes significantly in capturing attention. On the other hand, the results in Experiment 2 also suggest the involvement of individual-level visual properties in affecting pre-attentive face detection, since there was a highly significant main effect of identity.

## Experiment 3

In a between-subject design, this experiment tested how different amounts of prior experience with Mooney images affected search efficiency. Participants were divided into three groups: Group 1 was not given any prior information, Group 2 was given unambiguous conceptual information, and Group 3 was trained with Mooney images prior to participating in the present experiment, in addition to receiving the conceptual information given to Group 2.

### Methods

#### Participants

Twenty-nine students from Dartmouth College and the two authors (in total 14 females) participated in Experiment 3. All participants, except the authors, received course credit or were compensated for their time. All participants had normal or corrected to normal visual acuity and gave written, informed consent. These procedures were approved by the Committee for the Protection of Human Subjects at Dartmouth College and conducted in accordance with the 1964 Declaration of Helsinki.

#### Materials and Procedure

The procedure was the same as in Experiment 2 except: (1) three different Mooney faces were used for this experiment. All participants were tested with these Mooney faces both upright and inverted so there was no between participant comparison for the interaction of face identity and inversion; (2) For participants in Group 2 (*N* = 11) and Group 3 (*N* = 10), gray-scale versions of the Mooney face targets were shown alongside the target at the beginning of each block so participants had unambiguous prior knowledge about features and aspects of the target; (3) Group 3 included the two authors, who were most familiar with the Mooney stimuli. In this group, participants had additionally completed a separate study that involved learning to categorize 300 Mooney images as face/non-face over 7 days.

### Data Analysis

Accuracies were computed and RTs of correct trials were trimmed with the same criteria as Experiments 1 and 2. Next, to examine search efficiency, the slopes of the reaction time by set size were computed for each identity and each group separately. A four-way ANOVA was conducted with identity, inversion and target presence as within-subject factors and group as the between-subjects factor. The slope data were then separated by target presence and two three-way ANOVAs were conducted with identity and inversion as the within-subject factors and group as the between subject factors.

### Results

Accuracy rates ranged from 92.9 to 97.8% correct for upright targets and 93.3 to 96.6% correct for inverted targets. Accuracy was not significantly different between participant groups [*F*(2,28) = 0.76, *p* = 0.48, $\eta_{p}^{2}$ = 0.05], suggesting no speed–accuracy trade-offs.

**FIGURE 5:**
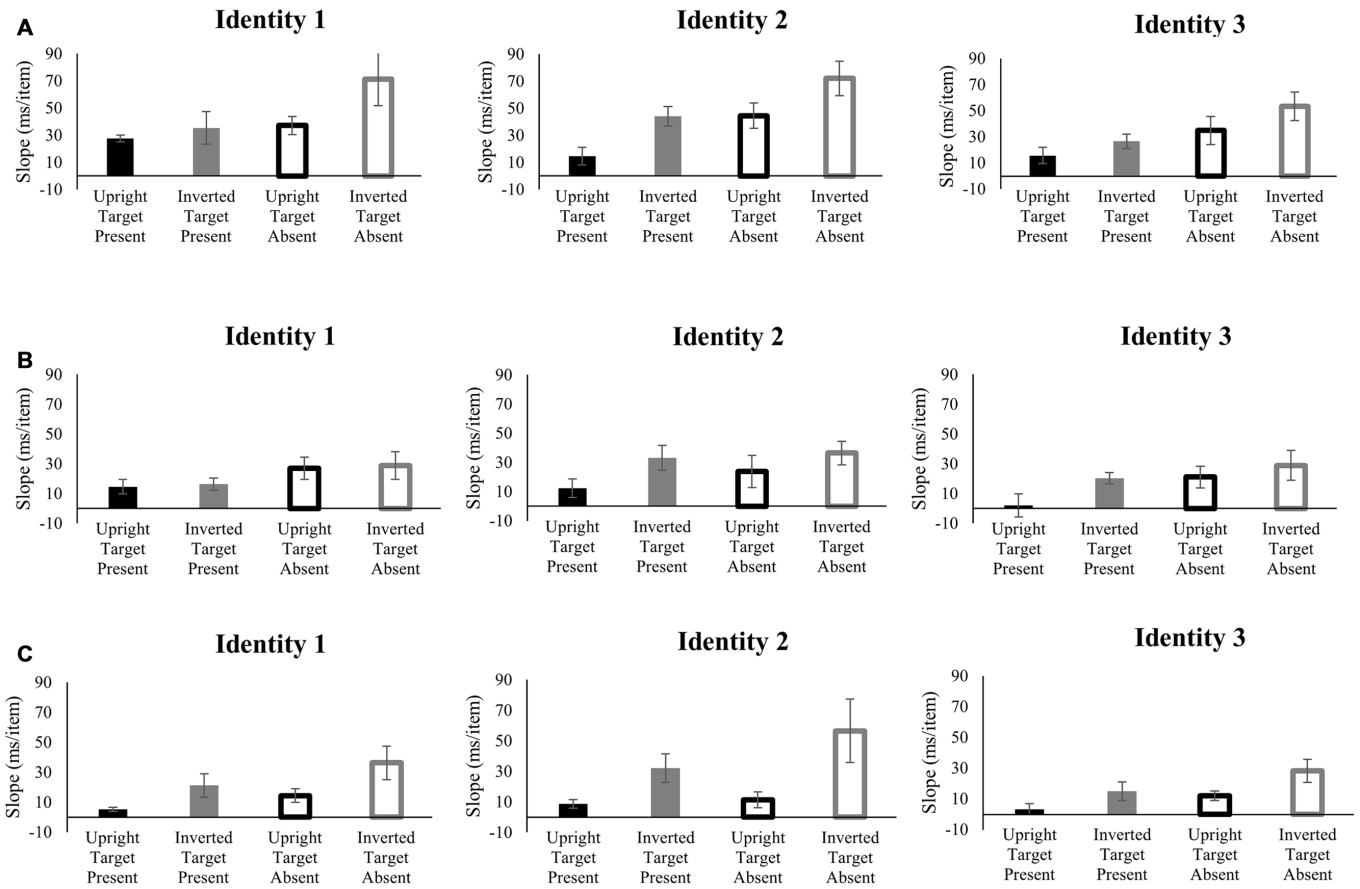
**Slopes of the reaction times as a function of search array set size for each target identity and each participant group in Experiment 3.** The efficiency of search for the Mooney face targets increased with more experience (A = Group 1, B = Group 2, C = Group 3). Black bar, upright; gray bar, inverted; filled bar, target-present; hollow bar, target-absent. Error bars represent ± 1 *SEM.*

The search reaction time slopes of each target identity by each group are shown in **Figure 5**. The four-way ANOVA on the slopes of reaction time by set size revealed significant main effects of target identity [*F*(2,56) = 5.23, *p* < 0.01, $\eta_{p}^{2}$ = 0.16], inversion [*F*(1,28) = 20.88, *p* < 10^-4^, $\eta_{p}^{2}$ = 0.43], target presence [*F*(1,28) = 29.16, *p* < 10^-5^, $\eta_{p}^{2}$ = 0.51] and group [*F*(2,28) = 6.53, *p* < 0.01, $\eta_{p}^{2}$ = 0.32]. No four-way, three-way, or two-way interactions were significant (*F* < 2.5, *p* > 0.05). *Post hoc* tests revealed that Group 1 was significantly different from both Group 2 and Group 3 (*p* < 0.05, Bonferroni corrected). However, Groups 2 and 3 were not significantly different from each other (*p* = 0.74). The three-way ANOVA performed on search efficiency slopes of target present trials also revealed significant main effects of target identity [*F*(2,56) = 4.93, *p* < 0.05, $\eta_{p}^{2}$ = 0.15], inversion [*F*(1,28) = 14.2, *p* < 0.001, $\eta_{p}^{2}$ = 0.34], and group [*F*(2,28) = 5.99, *p* < 0.01, $\eta_{p}^{2}$ = 0.30]. And the interaction between target identity and inversion was significant [*F*(2,56) = 3.72, *p* < 0.05, $\eta_{p}^{2}$ = 0.12]. *Post hoc* tests again revealed that Group 1 was significantly different from both Group 2 and Group 3 (*p* < 0.05, Bonferroni corrected), whereas Groups 2 and 3 were not significantly different from each other (*p* = 0.51). The three-way ANOVA performed on search efficiency slopes of target absent trials revealed significant main effects of inversion [*F*(1,28) = 17.0, *p* < 0.001, $\eta_{p}^{2}$ = 0.38] and group [*F*(2,28) = 5.24, *p* < 0.05, $\eta_{p}^{2}$ = 0.27]. And neither the main effect of target identity, nor any interaction was found to be significant for the target absent condition (*F* < 2.5, *p* > 0.05). *Post hoc* tests revealed that Group 1 was significantly different from Group 2 (*p* < 0.05, Bonferroni corrected), and marginally significantly different from Group 3 (*p* = 0.019, uncorrected). Again, Groups 2 and 3 were not significantly different from each other (*p* = 0.89).

### Discussion

By using Mooney images in visual search experiments we were able to investigate how different attributes of a face guide attention for rapid visual detection. Our results suggest that while upright gist information allows for more rapid face detection, Mooney images do not provide enough information on their own to facilitate pre-attentive detection. In Experiment 3, the combination of gist information, specific features and prior experience made Mooney faces capture attention in a similar manner as intact face pictures. Through merely analyzing image-level features, it is impossible to differentiate upright vs. inverted Mooney faces (Mooney, 1957). Nonetheless, participants were significantly more efficient in detecting upright Mooney faces than inverted Mooney faces. Participants were also more accurate in detecting upright Mooney faces than inverted Mooney faces, ruling out speed–accuracy trade-off. According to the reverse hierarchical theory of visual processing (Hochstein and Ahissar, 2002), search of upright Mooney faces should be efficient when gist is provided. Our results suggest a fine-tuning of this theory by revealing that efficient search of Mooney faces would also rely on both features of individual targets and prior experience of Mooney images.

As gist abstraction may include detecting whether there are holistic face patterns in the display (e.g., to differentiate social vs. non-social scenes), such processing does not necessarily require focused attention (Li et al., 2002; Rousselet et al., 2003; Evans and Treisman, 2005; Hershler and Hochstein, 2005; Furey et al., 2006; VanRullen, 2006; Palermo and Rhodes, 2007; Rossion and Caharel, 2011). Although visually degenerated, upright Mooney faces share the same gist with normal face pictures, and therefore, guided attention to enable more rapid detection in visual search. On the other hand, unlike pictures of faces, Mooney face targets are usually not searched efficiently (i.e., no ‘pop-out’ effects). In addition to whatever information remained in Mooney images that can be used to differentiate face vs. non-face as well as upright vs. inverted faces, low-level visual properties cannot be ruled out for affecting the spontaneous capturing of attention. Moreover, not all Mooney faces were searched with the same efficiency. This was revealed in Experiment 2 with the significant main effect of target identity. By using Mooney images, we equalized low-level features among the targets to a great extent (McKone, 2004). However, the significant main effect of identity and the significant interaction between identity and inversion suggest that the individual-level differences between targets still affect search efficiency. This result cannot be fully explained by the reverse hierarchical theory, since the significant effect of individual-level features contradicts that the gist could be processed solely at first. Because our stimuli were degenerated Mooney images, some of them may match a holistic/configural face pattern template for detection better than others (Farah et al., 1998). However, if a certain feature defined the target from distractors, the processing of faces would not necessarily precede the processing of specific features. Some information beyond what is presented in our Mooney images appeared to be necessary to differentiate face vs. non-face as rapidly as the previously reported efficiency for searching for pictures of faces (Hershler and Hochstein, 2005). In Experiment 3, different levels of conceptual information and experience were tested and significant differences were found between the tested groups independent of target identity. Moreover, *post hoc* analyses reveal that providing unambiguous face information (i.e., picture of face) rather than familiarity of Mooney images facilitated the search efficiency of Mooney face targets, suggesting that conceptual, top–down knowledge aids in how faces capture attention. In addition, the between-group effect was found to be significant in target-absent trials, revealing that top–down, experience driven information can also aid in the ability to quickly conclude that there is no face in a search display. The biased competition model of selective attention proposes that attention should not only facilitate the detection of targets but also suppress processing of distractors (Desimone and Duncan, 1995). While the recognition of Mooney images is heavily modulated by top–down effects of prior experience (Dolan et al., 1997; Hsieh et al., 2010; Gorlin et al., 2012), it appears that prior experience also helps to identify that a face is absent in a search display. Consistent with this notion, our results suggest that experience facilitates the gist extraction of Mooney face targets independently of target identity.

Given that participants in our Experiment 3 had, at most, a week of training with Mooney images, it remains possible that more training (such as a lifetimes worth) could lead to efficient search with all Mooney faces as well as enhanced effects of local-features. Note that the detection speed of about half of our upright Mooney face stimuli already fell below 10 ms/item in Experiments 2 and 3. The lack of detailed local visual features in Mooney images may explain why not all of the upright Mooney face targets were searched efficiently, but information from local visual features cannot be the main cause for rapid face detection, as discussed above. Then, how could it be possible that a Mooney face may readily capture attention? Cortical pathways starting from the primary visual cortex have been the main focus of vision research. However, additional subcortical pathways involving the superior colliculus, the pulvinar and the amygdala have been known to process visual information as well (Jones et al., 1976; Schiller and Malpeli, 1977; Tamietto and de Gelder, 2010). Neural responses through the cortical pathways are heavily modulated by attention (Kastner and Ungerleider, 2000). By contrast, implicit social and affective processing of face stimuli has been shown to involve the subcortical pathway, which is much faster (Whalen et al., 1998; Todorov et al., 2005). This pathway does not need to be modulated by attention (Whalen et al., 1998), therefore making it a possible route to explain efficient search for faces. In addition, recent eye-tracking studies revealed that saccades could be independent of perception (Lisi and Cavanagh, 2015). As face detection presumably occurs before any other face specific processing, visual search of faces and rapid saccades to faces may also share subcortical mechanisms, independent of the cortical processing of faces that leads to conscious but relatively slow perception. Future studies using neuroimaging techniques, such as EEG and fMRI, should provide further insights to understand the neural mechanisms underlying rapid face detection with Mooney images.

## Author Contributions

JG and MM designed the study. JG and MM conducted the study. JG and MM wrote the paper.

## Conflict of Interest Statement

The authors declare that the research was conducted in the absence of any commercial or financial relationships that could be construed as a potential conflict of interest.

## References

[B1] Brainard D. (1997). The psychophysics toolbox. *Spat. Vis.* 10 433–436. 10.1163/156856897X00357 9176952

[B2] Brown V. Huey D. Findlay J. M. (1997). Face detection in peripheral vision: do faces pop out? *Perception* 26 1555–1570. 10.1068/p261555 9616483

[B3] Carrasco M. Ponte D. Rechea C. Sampedro M. J. (1998). “Transient structures”: the effects of practice and distractor grouping on within-dimension conjunction searches. *Percept. Psychophys.* 60 1243–1258. 10.3758/BF03206173 9821785

[B4] Cauchoix M. Barragan-Jason G. Serre T. Barbeau E. (2014). The neural dynamics of face detection in the wild revealed by MVPA. *J. Neurosci.* 34 846–854. 10.1523/JNEUROSCI.3030-13.2014 24431443 PMC6608346

[B5] Crouzet S. Kirchner H. Thorpe S. (2010). Fast saccades toward faces: face detection in just 100ms. *J. Vis.* 10 1–17. 10.1167/10.4.16 20465335

[B6] Desimone R. Duncan J. (1995). Neural mechanisms of selective visual attention. *Annu. Rev. Neurosci.* 18 193–222. 10.1146/annurev.ne.18.030195.001205 7605061

[B7] Diamond R. Carey S. (1986). Why faces are and are not special: an effect of expertise. *J. Exp. Psychol. Gen.* 115 107–117. 10.1037/0096-3445.115.2.107 2940312

[B8] Doi H. Ueda K. (2007). Searching for a perceived stare in the crowd. *Perception* 36 773–780. 10.1068/p5614 17624121

[B9] Dolan R. Fink G. Rolls E. Booth M. Holmes A. Frackowiak R. (1997). How the brain learns to see objects and faces in an impoverished context. *Nature* 389 596–599. 10.1038/39309 9335498

[B10] Evans K. Treisman A. (2005). Perception of objects in natural scenes: is it really attention free? *J. Exp. Psychol. Hum. Percept. Perform.* 31 1476–1492. 10.1037/0096-1523.31.6.1476 16366803

[B11] Farah M. Wilson K. Drain M. Tanaka J. (1998). What is “special” about face perception? *Psychol. Rev.* 105 482–498. 10.1037/0033-295X.105.3.482 9697428

[B12] Farzin F. Rivera S. Whitney D. (2009). Holistic crowding of Mooney faces. *J. Vis.* 9 1–15. 10.1167/9.6.18 19761309 PMC2857385

[B13] Fine I. Wade A. R. Brewer A. A. May M. G. Goodman D. F. Boynton G. M. (2003). Long-term deprivation affects visual perception and cortex. *Nat. Neurosci.* 6 915–916. 10.1038/nn1102 12937420

[B14] Furey M. Tanskanen T. Beauchamp M. Avikainen S. Uutela K. Hair R. (2006). Dissociation of face-selective cortical responses by attention. *Proc. Natl. Acad. Sci. U.S.A.* 103 1065–1070. 10.1073/pnas.0510124103 16415158 PMC1348001

[B15] Gauthier I. Skudlarski P. Gore J. Anderson A. (2000). Expertise for cars and birds recruits brain areas involved in face recognition. *Nat. Neurosci.* 3 191–197. 10.1038/72140 10649576

[B16] Gauthier I. Tarr M. Anderson A. Skudlarski P. Gore J. (1999). Activation of the middle fusiform “face area” increases with expertise in recognizing novel objects. *Nat. Neurosci.* 2 568–573. 10.1038/9224 10448223

[B17] Gorlin S. Meng M. Sharma J. Sugihara H. Sur M. Sinha P. (2012). Imaging prior information in the brain. *Proc. Natl. Acad. Sci. U.S.A.* 109 7935–7940. 10.1073/pnas.1111224109 22538820 PMC3356663

[B18] Graham D. Meng M. (2011). Artistic representations: clues to efficient coding in human vision. *Vis. Neurosci.* 28 371–379. 10.1017/S095252381000162 21838937

[B19] Hershler O. Hochstein S. (2005). At first sight: a high-level pop out effect for faces. *Vision Res.* 45 1707–1724. 10.1016/j.visres.2004.12.021 15792845

[B20] Hochstein S. Ahissar M. (2002). View from the top: hierarchies and reverse hierarchies in the visual system. *Neuron* 36 791–804. 10.1016/S0896-6273(02)01091-7 12467584

[B21] Hsieh P. Vul E. Kanwisher N. (2010). Recognition alters the spatial pattern of fMRI activation in early retinotopic cortex. *J. Neurophysiol.* 103 1501–1507. 10.1152/jn.00812.2009 20071627 PMC3257064

[B22] Jiang Y. Costello P. He S. (2007). Processing of invisible stimuli: advantage of upright faces and recognizable words in overcoming interocular suppression. *Psychol. Sci.* 18 349–355. 10.1111/j.1467-9280.2007.01902.x 17470261

[B23] Johnson M. (2005). Subcortical face processing. *Nat. Rev. Neurosci.* 6 766–774. 10.1038/nrn1766 16276354

[B24] Jones E. Burton H. Saper C. Swanson L. (1976). Midbrain, diencephalic and cortical relationships of the basal nucleus of meynert and associated structures in primates. *J. Compar. Neurol.* 167 385–420. 10.1002/cne.901670402 818134

[B25] Kanwisher N. McDermott J. Chun M. (1997). The fusiform face area: a module in human extrastriate cortex specialized for face perception. *J. Neurosci.* 17 4302–4311. 9151747 10.1523/JNEUROSCI.17-11-04302.1997 PMC6573547

[B26] Kastner S. Ungerleider L. (2000). Mechanisms of visual attention in the human cortex. *Annu. Rev. Neurosci.* 23 315–341. 10.1146/annurev.neuro.23.1.315 10845067

[B27] Le Grand R. Mondloch C. J. Maurer D. Brent H. P. (2001a). Early visual experience and face processing. *Nature* 410:890 10.1038/35073749 11309606

[B28] Le Grand R. Mondloch C. J. Maurer D. Brent H. P. (2001b). Early visual experience and face processing: correction. *Nature* 412:786 10.1038/35090636 11309606

[B29] Li F. VanRullen R. Koch C. Perona P. (2002). Rapid natural scene categorization in the near absence of attention. *Proc. Natl. Acad. Sci. U.S.A.* 99 9596–9601. 10.1073/pnas.092277599 12077298 PMC123186

[B30] Lisi M. Cavanagh P. (2015). Dissociation between the perceptual and saccadic localization of moving objects. *Curr. Biol.* 25 1–6. 10.1016/j.cub.2015.08.021 26412133

[B31] Lorenzino M. Caudek C. (2015). Task-irrelevant emotion facilitates face discrimination learning. *Vision Res.* 108 56–66. 10.1016/j.visres.2015.01.007 25645963

[B32] Loschky L. Larson A. (2008). Localized information is necessary for scene categorization, including the natural/man-made distinction. *J. Vis.* 8 1–9. 10.1167/8.1.4 18318607

[B33] McKone E. (2004). Isolating the special component of face recognition: peripheral identification and a Mooney face. *J. Exp. Psychol. Learn. Mem. Cogn.* 30 181–197. 10.1037/0278-7393.30.1.181 14736306

[B34] Mooney C. (1957). Age in the development of closure ability in children. *Can. J. Psychol.* 11 219–226. 10.1037/0278-7393.30.1.181 13489559

[B35] Morton J. Johnson M. (1991). CONSPEC and CONLEARN: a two-process theory of infant face recognition. *Psychol. Rev.* 98 164–181. 10.1037/0033-295X.98.2.164 2047512

[B36] Nelson C. (2001). The development and neural bases of face recognition. *Infant Child Dev.* 10 3–18. 10.1002/icd.239

[B37] Oliva A. (2005). “Gist of the scene,” in *Neurobiology of Attention* eds Itti L. Rees G. Tsotsos J. (San Diego, CA: Elsevier) 251–256.

[B38] Ostrovsky Y. Andalman A. Sinha P. (2006). Vision following extended congenital blindness. *Psychol. Sci.* 17 1009–1014. 10.1111/j.1467-9280.2006.01827.x 17201779

[B39] Palermo R. Rhodes G. (2007). Are you always on my mind? A review of how face perception and attention interact. *Neuropsychologia* 45 75–92. 10.1016/j.neuropsychologia.2006.04.025 16797607

[B40] Rossion B. Caharel S. (2011). ERP evidence for the speed of face categorization in the human brain: disentangling the contribution of low-level visual cues from face perception. *Vision Res.* 51 1297–1311. 10.1016/j.visres.2011.04.003 21549144

[B41] Rousselet G. Mace M. Fabre-Thrope M. (2003). Is it an animal? Is it a human face? Face processing in upright and inverted natural scenes. *J. Vis.* 3 440–455. 10.1167/3.6.5 12901715

[B42] Schiller P. Malpeli J. (1977). Properties and tectal projections of monkey retinal ganglion cells. *J. Neurophysiol.* 40 428–445. 403252 10.1152/jn.1977.40.2.428

[B43] Tamietto M. de Gelder B. (2010). Neural bases of the non-conscious perception of emotional signals. *Nat. Rev. Neurosci.* 11 697–709. 10.1038/nrn2889 20811475

[B44] Todorov A. Mandisodza A. Goren A. Hall C. (2005). Inferences of competence from faces predict election outcomes. *Science* 308 1623–1626. 10.1126/science.1110589 15947187

[B45] Tong F. Nakayama K. (1999). Robust representations for faces: evidence from visual search. *J. Exp. Psychol. Hum. Percept. Perform.* 25 1016–1035. 10.1037/0096-1523.25.4.1016 10464943

[B46] Treisman A. Gelade G. (1980). A feature-integration theory of attention. *Cogn. Psychol.* 12 97–136. 10.1016/0010-0285(80)90005-5 7351125

[B47] VanRullen R. (2006). On second glance: still no high-level pop-out effect for faces. *Vision Res.* 46 3017–3027. 10.1016/j.visres.2005.07.009 16125749

[B48] Wang Q. Cavanagh P. Green M. (1994). Familiarity and pop-out in visual search. *Percept. Psychophys.* 56 495–500. 10.3758/BF03206946 7991347

[B49] Whalen P. Rauch S. Etcoff N. McInerney S. Lee M. Jenike M. (1998). Masked presentations of emotional facial expressions modulate amygdala activity without explicit knowledge. *J. Neurosci.* 18 411–418. 9412517 10.1523/JNEUROSCI.18-01-00411.1998 PMC6793390

[B50] Willenbockel V. Sadr J. Fiset D. Horne G. Gosselin F. Tanaka J. (2010). Controlling low-level image properties: the SHINE toolbox. *Behav. Res. Methods* 42 671–684. 10.3758/BRM.42.3.671 20805589

[B51] Williams M. Moss S. Bradshaw J. Mattingley J. (2005). Look at me, I’m smiling: visual search for threatening and nonthreatening facial expressions. *Vis. Cogn.* 12 29–50. 10.1080/13506280444000193

[B52] Wolfe J. (1998). What can 1 million trials tell us about visual search? *Psychol. Sci.* 9 33–39. 10.1037/xhp0000012

[B53] Yang H. Gors J. Meng M. (2011). Face-semblance leads to faster visual search and breaking interocular suppression. *J. Vis.* 11:661 10.1167/11.11.661

[B54] Yang H. Zelinsky G. (2009). Visual search is guided to categorically-defined targets. *Vision Res.* 49 2095–2103. 10.1016/j.visres.2009.05.017 19500615 PMC2756560

